# The Efficacy of Inpatient vs. Home-Based Physiotherapy Following Coronary Artery Bypass Grafting

**DOI:** 10.3390/ijerph15112572

**Published:** 2018-11-17

**Authors:** Aleksandra Szylińska, Mariusz Listewnik, Iwona Rotter, Aleksandra Rył, Katarzyna Kotfis, Krzysztof Mokrzycki, Ewelina Kuligowska, Paweł Walerowicz, Mirosław Brykczyński

**Affiliations:** 1 Department of Medical Rehabilitation and Clinical Physiotherapy, Pomeranian Medical University in Szczecin, ul. Żołnierska 54, 70-204 Szczecin, Poland; aleksandra.szylinska@gmail.com (A.S.); iwona.rotter@pum.edu.pl (I.R.); 2 Department of Cardiac Surgery, Pomeranian Medical University in Szczecin, al. Powstańców Wlkp. 72, 70-111 Szczecin, Poland; sindbaad@poczta.onet.pl (M.L.); chrismo9.km@gmail.com (K.M.); ewelinabkulig@gmail.com (E.K.); pawel.walerowicz@gmail.com (P.W.); mirobryk@pum.edu.pl (M.B.); 3 Department of Anesthesiology, Intensive Therapy and Acute Intoxications, Pomeranian Medical University in Szczecin, al. Powstańców Wlkp. 72, 70-111 Szczecin, Poland; k.kotfis@wp.pl

**Keywords:** cardiac rehabilitation, forced expiratory volume in 1 s, forced vital capacity, coronary artery bypass grafting

## Abstract

*Background*: Intensive post-operative physiotherapy after cardiac surgery helps to reduce the number of complications, accelerating convalescence and decreasing peri-operative mortality. Cardiac rehabilitation is aimed at regaining lost function and sustaining the effect of cardiac surgery. The aim of this study was to compare the efficacy of inpatient and home-based phase II physiotherapy following coronary artery bypass grafting, and inpatient phase II post-operative physiotherapy based on the analysis of the spirometry results. *Methods*: A prospective observational study included 104 adult patients of both sexes undergoing planned coronary artery bypass grafting and were randomized to one of the two groups—inpatients (InPhysio) and home-based (HomePhysio) at a 1:1 ratio. All patients had undergone spirometry testing prior to surgery (S1) and on the fifth day after the operation (S2), i.e., on the day of completion of the first phase (PI) of physiotherapy. Both the study group (InPhysio) and the control group (HomePhysio) performed the same set of exercises in the second phase (PII) of cardiac physiotherapy, either in the hospital or at home, respectively, according to the program obtained in the hospital. Both groups have undergone spirometry testing (S3) at 30 days after the operation. *Results*: The demographic and peri-operative data for both groups were comparable and showed no statistically significant differences. An analysis of gradients between the results of spirometry tests before surgery and at 30 days after the surgery showed a smaller decrease in forced vital capacity (FVC) in the study group than in the control group (*p* < 0.001). The results at five and 30 days after the surgery showed a greater increase in FVC in the study group than in the control group (680 mL vs. 450 mL, *p* = 0.009). There were no statistically significant differences in other parameters studied. *Conclusions*: The advantage of inpatient over home-based physiotherapy was evidenced by much smaller decreases in FVC between the initial and final tests, and greater increases between the fifth day after surgery and the final test. Our analysis showed greater efficacy of inpatient physiotherapy as compared with home-based exercises and raises concerns about patient adherence.

## 1. Introduction

Advancements in modern treatment methods, including interventional cardiology and cardiac surgery, have resulted in a reduction in mortality from coronary artery disease [1,2,3,4]. In recent years, patients who are more burdened, older, with many co-morbidities and higher peri-operative risks, are qualifying for cardiac surgery. Currently, almost 60% of operations are performed in patients over 65 years of age [5,6]. Despite improvement in surgical and anesthetic techniques, cardiac surgery still imposes a huge burden on the patient’s homeostasis. Extracorporeal circulation and mechanical ventilation may have an adverse effect and contribute to the occurrence of peri-operative complications [2]. The first 24 hours after cardiac surgery are accompanied by a reduction in the efficiency of the respiratory system, including a decrease in functional residual capacity and forced vital capacity [7]. Acute respiratory failure, causing prolonged mechanical ventilation, is diagnosed in 0.4% to 2% of patients after cardiac surgery. Most complications are associated with respiratory infections [8]. The risk of death is increased by 50% in patients with prolonged ventilation or repeated use of mechanical ventilation [8,9,10].

In Poland, as in many other countries, the post-cardiac surgery rehabilitation process has three stages. The first phase is inpatient hospital physiotherapy, the second phase can be performed either as inpatient hospital-based physiotherapy or outpatient and home-based physiotherapy and the third phase is entirely outpatient or home-based physiotherapy [11]. A three-phase rehabilitation process is aimed at regaining lost function and sustaining the effect of cardiac surgery. Phase I of rehabilitation occurring in the hospital is designed to mobilize the patient as soon as possible and to help the patient regain independence in daily life activities. Phase II should include aggressive education and secondary prevention of cardiovascular events, and provide exercise monitoring. Phase III is a continuation of exercises learned at earlier stages and further reduction of cardiovascular risk factors [12,13,14].

Spirometry tests performed before surgery provide measurable data regarding the functioning of the respiratory system, informing about the occurrence of respiratory disorders and indicating potentially increased risk of complications in the early post-operative period. The studies have shown that spirometry performed prior to and after the surgery can be regarded as a useful and repeatable tool to provide information about the status of the respiratory system’s capacity and function [15,16,17]. The use of methods allowing an accurate analysis of the respiratory system before and after surgery makes it possible to design individual physiotherapy plans, reducing the number of pulmonary complications after surgery and qualifying the patients for certain cardiac rehabilitation processes. The aim of this study was to compare the efficacy of inpatient and home-based phase II physiotherapy following coronary artery bypass grafting, and inpatient phase II post-operative physiotherapy based on the analysis of the spirometry results.

## 2. Material and Methods

This prospective observational study included 104 adult patients (males and females) eligible for a planned coronary artery bypass graft, operated on between January 2015 and December 2016 in a tertiary reference university hospital. The study exclusion criteria were: emergency surgery, lack of consent, contraindications to perform spirometry testing and inability to perform maximum exhalation, as well as patients who were unable to perform the test correctly. Patients were randomized to one of the two groups—inpatients (InPhysio) and home-based (HomePhysio) at a 1:1 ratio. After surgery, patients selected for inpatient phase II (PII) physiotherapy (*n* = 52) were paired with a suitable patient from the group selected for home-based phase II rehabilitation meeting identical or similar criteria in terms of sex, age (below 65 and above 65), BMI (body mass index) (less than 30 kg/m^2^ and above 30 kg/m^2^) and coexisting medical conditions (hypertension, diabetes, chronic obstructive lung disease, myocardial infarction) (*n* = 52).

The research was carried out in accordance with the requirements of the Helsinki Declaration. All patients provided written informed consent to participate in the study and were informed about the course and the purpose of the study. All patients also consented to the use and processing of their medical data for the study, which was approved by the Bioethics Committee (KB-0012/121/14).

### 2.1. Pre-Operative Data

Before the operations, interviews were used to collect the data from the patients regarding co-morbidities and previous history of hypertension, diabetes, angina pectoris, myocardial infarctions, atrial fibrillation, heart failure, kidney disease, cancer, neurological diseases, orthopedic disorders and chronic obstructive pulmonary disease.

A peri-operative risk assessment was calculated using the EuroScore Logistics II (ESL) scale. Blood pressure, body mass and height were measured in all patients. Based on the anthropometric measurements, a BMI index was calculated for each patient using the standard formula: BMI = body mass (kg)/height^2^ (m). A normal body mass was defined as BMI in the range of 18.5–24.99 kg/m^2^, overweight in the range of 25–29.99 kg/m^2^, and obese for BMI ≥ 30 kg/m^2^. Demographic data is presented in Table 1. A list of post-operative complications in both groups is shown in Table 2.

### 2.2. Treatment and Principles of Applied Physiotherapy

All patients underwent planned isolated coronary artery bypass grafting with extracorporeal circulation via median sternotomy, and remained in the post-operative intensive care unit and cardiac surgery ward after the operation. They were weaned off mechanical ventilation once they met extubation criteria according to a local protocol (on average after ten hours). Phase I (P1) cardiac rehabilitation was initiated in the pre-operative period and continued after surgery and in the cardiac surgery ward.

Patients qualified for inpatient physiotherapy (InPhysio) trained on cycloergometers and had collective gym exercises in the rehabilitation room. Cycloergometer workouts were held twice a day from Monday to Saturday, 2 h after meals, under the supervision of physiotherapists. The load and type of training (with intervals or continuous) were adapted individually to the patient’s condition. Sessions on cycloergometers took about 20–30 min depending on the capability and strength of the patient. General gymnastics lasted 20 min each day, and breathing exercises with the use of TriFlo (Plastimed, Istanbul, Turkey) were performed 10 times every hour. The study group underwent physiotherapy at the Cardiac Physiotherapy Ward for about three weeks. In addition to pre-scheduled stationary exercises, patients were going for walks outside in good weather conditions.

The control group (HomePhysio) selected for home-based rehabilitation received detailed rehabilitation recommendations, handbooks and brochures on post-operative physiotherapy conduct and the performance of appropriate exercises, and were covered by outpatient care at the Cardiac Surgery Outpatient Clinic.

### 2.3. Spirometry Test

Patients that qualified for the study had spirometry tests on the day of admission to the hospital (S1), on the fifth day after surgery (S2) and 30 days after surgery (S3), using AsSPIRO D200 v.101 instruments (ASPEL SA, Zabierzow, Poland) and performed by a trained technician. Each patient was thoroughly instructed about the technique and the course of the study. For safety reasons, spirometry tests were performed in a sitting position. The tests were carried out in accordance with the recommendations of The American Thoracic Society (ATS) and The European Respiratory Society (ERS) [18,19,20].

### 2.4. Statistical Analysis

Statistical analysis was performed using Statistica 12.0 software (StatSoft, Inc., Tulsa, OK, USA). Descriptive statistics were used to characterize the group: means and standard deviations. A Shapiro–Wilk test was used to assess the normality of the distribution of the studied variables. The homogeneity of variance was carried out using the Levene test. In almost all datasets, the distribution deviated from normal or homogeneity of variance; therefore, non-parametric tests were used to analyze quantitative data. Evaluation of differences (gradients) in spirometry test results was performed using a Mann-Whitney U test. The chi-squared test was used to analyze the qualitative data, and for a low number in the subgroup, a Yates correction or Fisher’s exact test was used. To assess the differences between the results of spirometry tests conducted before surgery, at five and 25–30 days after surgery, a Wilcoxon signed rank test for dependent variables was used. A significance level of *p* ≤ 0.05 was assumed.

### 2.5. Characteristics of Patients

Table 1 presents the data collected on the day of admission to the Cardiac Surgery Department. The study and control groups did not differ significantly in terms of selected features.

Table 2 presents the type and frequency of post-operative complications in both groups. The most frequent complication was atrial fibrillation. There were no significant differences between the test and control groups.

## 3. Results

**Figure 1:**
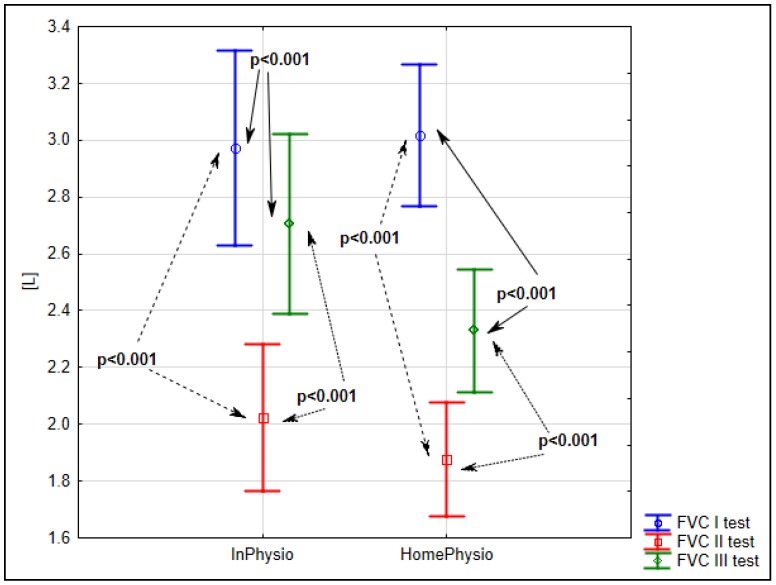
Evaluation of mean FVC before surgery (S1), five (S2) and 25–30 days after surgery (S3) in the InPhysio and HomePhysio groups.

Analysis of the results of spirometry tests before surgery (S1), and at five (S2) and 30 days (S3) after surgery showed that the mean value of forced vital capacity (FVC) had significantly decreased on the fifth day after the surgery, down to 68% of the admission level (*p* < 0.001). At 30 days after surgery, FVC had improved to 91% of the admission level (*p* < 0.001) for the inpatient study group (InPhysio) and to 77% (*p* < 0.001) for the home-care control group (HomePhysio). The detailed data is shown in Figure 1.

**Figure 2:**
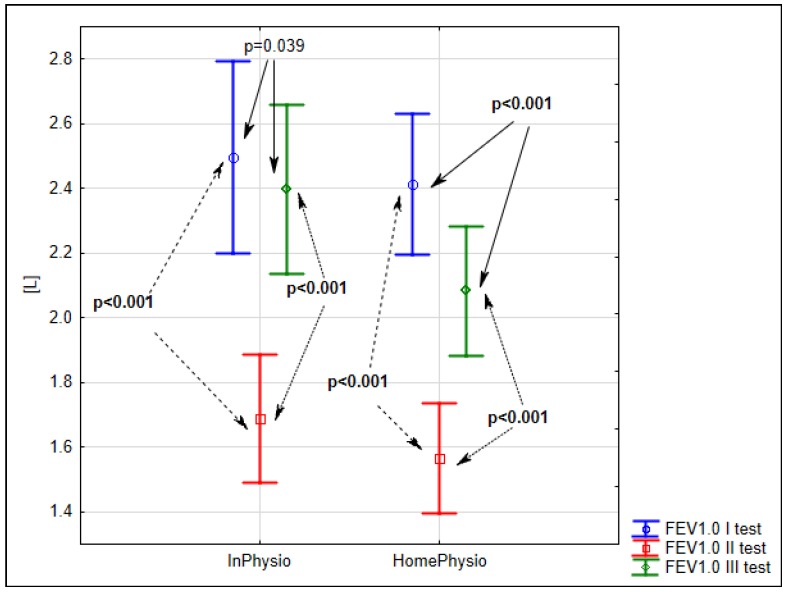
Evaluation of mean FEV 1.0 before surgery (S1), five (S2) and 25–30 days after surgery (S3) in the InPhysio and HomePhysio groups.

The next major spirometric parameter was forced expiratory volume in one second (FEV 1.0), which had also decreased on the fifth day after surgery to 68% of the admission level (*p* < 0.001) for the inpatient study group and to 65% (*p* < 0.001) for the home-care control group. At 30 days after surgery, FEV 1.0 had improved to 96% of the admission level (*p* = 0.039) for the inpatient study group and to 86% (*p* < 0.001) for the home-care control group (Figure 2).

The other parameters evaluated in our research included the Tiffeneau–Pinelli index (FEV1/FVC), peak expiratory flow (PEF) and maximum expiratory flow (MEF) at selected intervals of forced exhalation: 25%, 50% and 75% (MEF25, MEF50, and MEF75). The Tiffeneau–Pinelli index increased in both groups in each subsequent test. In the intra-group assessment, significance was achieved between the results of the S1 and S3 InPhysio (*p* = 0.019), HomePhysio (*p* < 0.001) and S2 and S3 tests InPhysio (*p* = 0.05), HomePhysio (*p* = 0.004).

In both groups, a significant decrease in mean PEF values was observed on the fifth day after surgery, followed by an increase at 30 days after surgery to a level close to the admission test. In the intra-group assessment, significant differences (*p* < 0.001) were achieved between the results of the S1 and S2, and S2 and S3 tests in the In Physio and HomePhysio groups. The differences between the baseline and final results were not significant (*p* = 0.94 and *p* = 0.81). MEF levels behaved similar to PEF.

Our analysis also included intergroup comparisons. The mean values of the results of FVC and FEV in the study and control groups before surgery, on the fifth day after surgery and on day 30 after the operation did not differ between the groups. Therefore, in further studies, a dynamic evaluation of the results was used, focusing on individual differences (gradients) in the measurement results between the tests before surgery and on the fifth and 30th days after surgery, and between five and 25–30 days after surgery.

Analysis of the gradients between the results of spirometric tests before surgery and on the fifth post-operative day showed a decrease in the value of all results in the test and control groups, except for the Tiffeneau index gradient in both groups. Differences between the groups were not statistically significant on the same days of the tests. However, when it comes to changes in time (gradients), in the inpatient rehabilitation group, the results of the final FVC level was only 270 mL lower than the baseline, while in the home-based rehabilitation group, it was 690 mL lower than baseline (*p* < 0.001). A gradient analysis of FVC between the tests carried out five and 30 days after surgery showed statistically significant differences of FVC values between the study group and the control group (680 mL vs. 450 mL, *p* = 0.009). Other parameters showed no statistically significant differences. Data are presented in Table 3.

## 4. Discussion

Numerous scientific studies confirm the effectiveness of cardiac rehabilitation in the reduction of post-operative mortality, where safe and effective exercise increases physical fitness, improves the quality of life, and also reduces the occurrence of complications and subsequent cardiovascular incidents [21,22,23,24,25,26,27,28,29,30,31,32,33]. In this study, we have shown statistically significant differences in FVC gradients between final and baseline results, as well as between the final results and those obtained five days after surgery between inpatient and home-based rehabilitation groups. Although neither group returned to pre-operative FVC levels, the advantage of inpatient rehabilitation was clear. The differences in final FEV 1.0 were not significant between the groups, but the inpatient rehabilitation patients had recovered to 96% of baseline, while in the control group, it was 86%.

Piepoli et al. describes cardiovascular physiotherapy as the core that promotes better quality of life and helps control risk factors for cardiovascular diseases [22]. It is believed that even a short-term cardiological rehabilitation program, combined with educational meetings on changing eating habits, is beneficial in modifying lifestyle [34,35,36]. An analysis of the benefits of home-based rehabilitation was carried out by Kim, who showed that 78% of patients had no awareness of the existence of rehabilitation programs, and patients often refused to participate in inpatient rehabilitation due to a lack of faith in its effectiveness, lack of time, anxiety, high costs or poor physical condition, as well as the poor availability of rehabilitation centers related to considerable distance or communication problems. Kim showed that although the results of inpatient rehabilitation were better than home-based rehabilitation, an improvement could be observed in either group [37].

Peri-operative spirometry may be important in monitoring respiratory failure associated with surgery, especially in monitoring the progress of rehabilitation, because it provides measurable information about the level of respiratory system abnormalities. The result of a functional respiratory test providing information about lung function disorders before surgery may contribute to a faster and more effective respiratory system improvement after surgery and may be important in preventing complications after surgical heart revascularization [15]. It is worth noting that the functioning of the respiratory system examined with a spirometer allows for the assessment of skeletal muscle strength as well as the velocity of airflow through the airways [38]. McAllister assessed the usefulness of forced expiratory volume in one second (FEV 1.0) as an indicator of the possible increase in mortality after cardiac surgery and longer patient stay at the hospital. In his opinion, FEV 1.0 should be added to the current EuroScore operational risk assessment scale due to the availability and ease of spirometric testing [39]. An analysis of the works that used pre- and post-operative spirometry tests shows a significant decrease in the value of all measured parameters. It does not really matter when exactly in the early post-operative period the test was carried out, because the results of tests performed on the third, fourth, fifth or seventh post-operative days showed a significant reduction in FVC, FEV 1.0, MEF and PEF. Only the Tiffeneau index (FEV 1.0 divided by FVC) increased in some studies [40,41,42,43,44,45,46,47,48]. Similar results were obtained on the fifth day after surgery in this study. Both the study group (inpatient rehabilitation) and control group (home-based rehabilitation) showed a decrease in FVC to 68% and 62% of baseline levels, and FEV 1.0 to 68% and 65% of baseline. Differences between the results of both groups were not significant.

There are only a few works that used a similar research method in the assessment of various types of post-surgical rehabilitation. Westerdahl examined 98 men who had undergone coronary artery bypass grafting. The first group performed breathing exercises with resistance exhalation into a water bottle, the second group exercised using the IR-PEEP (inspiratory resistance-positive expiratory pressure mask) mask, while the third group performed normal breathing exercises without additional equipment. In all groups, these spirometry tests showed a significant decrease between the day of surgery and the fourth day after surgery. Differences between the results of all groups were not statistically significant, except for the total lung capacity (TLC), which was significantly lower in the group performing resistance exercise with the bottle compared to the patients exercising without any equipment [49]. Peijl analyzed the results of a seven-day hospital rehabilitation following CABG (coronary artery bypass graft). Instead of spirometry, the comparison of inpatient and home-based rehabilitation relied on the measurement of the time to achieve five goals (switching from bed to chair, walking around the room, walking in the ward, participating in group exercises and climbing stairs). Slightly better results, and only partially statistically significant, were obtained in the group undergoing intensive inpatient rehabilitation [50].

Shakuri presented a comparative analysis of two groups of patients after surgical heart revascularization, in which a study group underwent 15 days of pre-operative physiotherapy and standard post-operative rehabilitation, while in the control group, only the latter was used. In the assessment of the results, the march test was used in addition to spirometry. Significantly better results were obtained in the study group [51]. A slightly different approach was presented by Spirowski analyzing post-operative recovery using a cycloergometer. Only one group of patients with intensive rehabilitation after CABG was evaluated, and control examinations after three weeks and six months showed a statistically significant improvement compared to the pre-operative examination [52].

## 5. Study Limitations

Our study was limited by the number of patients with particular types of respiratory disorders. In order to obtain more representative results for the general population, the number of patients should be increased in future research. The sociodemographic information regarding the education level or the housing background were not collected, and these might be important factors when qualifying the patients for home-based physiotherapy. In the control group with home-based rehabilitation, we did not use regular telephone reminders regarding physical exercises. Therefore, there is a risk that some patients did not comply with the process at all.

## 6. Conclusions

The advantage of inpatient over home-based physiotherapy was evidenced by much smaller decreases in FVC between the initial and final tests, and greater increases between the fifth day after surgery and the final test. Our analysis showed greater efficacy of inpatient physiotherapy as compared with home-based exercises and raises concerns about patient adherence.

## Figures and Tables

**Table 1:** Characteristics of selected pre-, intra- and post-operative parameters in both groups.

Variables		InPhysio (*n* = 52)	HomePhysio (*n* = 52)	*p*-Value
Demographic data				
Age ($\bar{x}$ ± SD, years)		64.23 ± 8.31	65.06 ± 7.89	0.329
Sex	Women (*n* = 41)	19 (36.54%)	22 (42.31%)	0.547 *
	Men (*n* = 63)	33 (63.46%)	30 (57.69%)	
BMI ($\bar{x}$ ± SD, kg/m^2^)		30.12 ± 5.00	29.73 ± 4.35	0.881
Smoking	Smokers (*n* = 36)	20 (38.46%)	16 (30.77%)	0.409 *
	($\bar{x}$ ± SD, years)	29.62 ± 12.16	35.00 ± 10.69	0.259
Operative risk				
EuroScore II ($\bar{x}$ ± SD, %)		4.28 ± 4.80	4.87 ± 3.70	0.072
Pre-operative data				
Duration of the underlying disease ($\bar{x}$ ± SD, months)		47.85 ± 66.20	49.76 ± 63.36	0.996
EF ($\bar{x}$ ± SD, %)		48.08 ± 8.70	47.37 ± 8.40	0.666
CKMB ($\bar{x}$ ± SD, IU/L)		22.10 ± 9.54	24.07 ± 9.59	0.221
Glycated hemoglobin ($\bar{x}$ ± SD, %)		6.28 ± 1.02	6.35 ± 1.06	0.691
Creatinine ($\bar{x}$ ± SD, mg/dL)		0.86 ± 0.24	0.92 ± 0.51	0.647
GFR ($\bar{x}$ ± SD, mL/min/1.73 m^2^)		84.51 ± 16.01	83.98 ± 16.16	0.500
CRP ($\bar{x}$ ± SD, mg/L)		4.63 ± 9.89	2.67 ± 3.81	0.097
Intra-operative data				
Perfusion time ($\bar{x}$ ± SD, min)		49.02 ± 11.04	53.25 ± 11.34	0.070
Aortic clamping time ($\bar{x}$ ± SD, min)		29.69 ± 9.46	32.71 ± 8.42	0.056
Post-operative data				
Intubation time ($\bar{x}$ ± SD, min)		634.12 ± 234.29	689.81 ± 253.63	0.199
CKMB ($\bar{x}$ ± SD, IU/L)		42.35 ± 16.28	47.58 ± 18.55	0.124
Creatinine max ($\bar{x}$ ± SD, mg/dL)		0.99 ± 0.38	1.07 ± 0.65	0.762
GFR min ($\bar{x}$ ± SD, mL/min/1.73 m^2^)		77.67 ± 21.22	76.25 ± 22.51	0.908
CRP—second day ($\bar{x}$ ± SD, mg/L)		64.61 ± 31.42	62.16 ± 36.29	0.484
CRP—fourth day ($\bar{x}$ ± SD, mg/L)		220.82 ± 67.91	223.45 ± 75.14	0.964

$\bar{x}$—mean, SD—standard deviation, *p*—statistical significance, *n*—number of patients, EF—ejection fraction, CKMB—creatine kinase-MB, GFR—glomerular filtration rate, CRP—C-reactive protein, FFP—fresh frozen plasma, PRBCT—packed red blood cell transfusion. Note: calculations of *p* based on the exact chi-squared test (*) or the Mann-Whitney U-test.

**Table 2:** A list of post-operative complications in both groups.

Post-Operative Complications	InPhysio (*n* = 52)	HomePhysio (*n* = 52)	*p*-Value
	*n* (%)	*n* (%)	
Acute renal failure treated with hemofiltration	0 (0.00%)	1 (1.92%)	0.500
Re-intubation	1 (1.92%)	3 (5.77%)	0.618
Hydrothorax/hemothorax requiring drainage	3 (5.77%)	3 (5.77%)	0.661
Post-operative delirium	3 (5.77%)	3 (5.77%)	0.661
Re-operation due to bleeding or tamponade	0 (0.00%)	1 (1.92%)	0.500
Atrial fibrillation	5 (9.61)	9 (17.31%)	0.250
Sternal wound infection	1 (1.92%)	4 (7.69%)	0.363
Infection after endoscopic vein harvest	2 (3.85%)	2 (3.85%)	0.691
Sternum instability requiring intervention	0 (0.00%)	1 (1.92%)	0.500

*p*—statistical significance, *n*—number of patients. Note: calculations of *p* based on the exact chi-squared test with Yates correction.

**Table 3:** The intra-group assessment. Evaluation of average gradients of spirometry test results using means and standard deviations.

Gradients of Spirometry Tests		InPhysio (*n* = 52)	HomePhysio (*n* = 52)	*p*-Value
		Mean ± SD	Mean ± SD	
Gradient of S1 and S2 spirometry tests	FVC (L)	−0.95 ± 0.61	−1.14 ± 0.6	NS
	FEV 1.0 (L)	−0.81 ± 0.74	−0.85 ± 0.58	NS
	PEF (L/s)	−1.34 ± 1.98	−1.54 ± 2.23	NS
Gradient of S1 and S3 spirometry tests	FVC (L)	−0.27 ± 0.57	−0.69 ± 0.51	<0.001
	FEV 1.0 (L)	−0.10±0.65	−0.33 ± 0.57	NS
	PEF (L/s)	−0.17 ± 2.24	−0.18 ± 2.56	NS
Gradient of S2 and S3 spirometry tests	FVC (L)	0.68 ± 0.54	0.45 ± 0.47	0.009
	FEV 1.0 (L)	0.71 ± 0.55	0.52 ± 0.46	NS
	PEF (L/s)	1.17 ± 2.21	1.36 ± 1.57	NS

*n*—number of patients, SD—standard deviation, *p*—statistical significance, L—liters, s—seconds Note: calculations of *p* based on the exact the Mann-Whitney U-test.
